# ﻿20 years of bibliometric data illustrates a lack of concordance between journal impact factor and fungal species discovery in systematic mycology

**DOI:** 10.3897/mycokeys.110.136048

**Published:** 2024-11-20

**Authors:** R. Henrik Nilsson, Arnold Tobias Jansson, Christian Wurzbacher, Sten Anslan, Pauline Belford, Natàlia Corcoll, Alexandra Dombrowski, Masoomeh Ghobad-Nejhad, Mikael Gustavsson, Daniela Gómez-Martínez, Faheema Kalsoom Khan, Maryia Khomich, Charlotte Lennartsdotter, David Lund, Breyten Van Der Merwe, Vladimir Mikryukov, Marko Peterson, Teresita M. Porter, Sergei Põlme, Alice Retter, Marisol Sanchez-Garcia, Sten Svantesson, Patrik Svedberg, Duong Vu, Martin Ryberg, Kessy Abarenkov, Erik Kristiansson

**Affiliations:** 1 Department of Biological and Environmental Sciences, Gothenburg Global Biodiversity Centre, University of Gothenburg, Box 463, 405 30 Göteborg, Sweden; 2 Chair of Urban Water Systems Engineering, Technical University of Munich, Am Coulombwall 3, 85748 Garching, Germany; 3 Department of Biological and Environmental Science, University of Jyväskylä, Survontie 9, 40014 Jyväskylän yliopisto, Finland; 4 Institute of Ecology and Earth Sciences, University of Tartu, Liivi 2, 50409 Tartu, Estonia; 5 Department of Computer Science and Engineering, Interaction Design, University of Gothenburg, Lindholmsplatsen 1, 41756 Göteborg, Sweden; 6 Department of Biological and Environmental Sciences, FRAM Centre for Future Chemical Risk Assessment and Management Strategies, University of Gothenburg, Box 463, 405 30 Göteborg, Sweden; 7 Department of Biotechnology, Iranian Research Organization for Science and Technology (IROST), P. O. Box 3353-5111, Tehran, Iran; 8 Systematic Biology, Department of Organismal Biology, Evolutionary Biology Center, Uppsala University, Norbyvägen 18 D, 752 36 Uppsala, Sweden; 9 Department of Clinical Science, University of Bergen, Box 7804, 5020 Bergen, Norway; 10 Department of Mathematical Sciences, Chalmers University of Technology and University of Gothenburg, Göteborg, Sweden; 11 Department of Chemical Engineering, Stellenbosch University, Private Bag X1, Stellenbosch, South Africa; 12 Toronto, Ontario, Canada; 13 Department of Functional and Evolutionary Ecology, University of Vienna, Djerassiplatz 1, A-1030 Vienna, Austria; 14 Leibniz Institute for Freshwater Ecology and Inland Fisheries, IGB, Zur alten Fischerhuette 2, 16775 Neuglobsow, Germany; 15 Department of Forest Mycology and Plant Pathology, Swedish University of Agricultural Sciences, Box 7026, 750 07 Uppsala, Sweden; 16 Westerdijk Fungal Biodiversity Institute, Uppsalalaan 8, 3584 CT Utrecht, Netherlands; 17 Natural History Museum, University of Tartu, Vanemuise 46, Tartu 51014, Estonia

**Keywords:** Bibliometrics, impact factor, mycology, systematics, taxonomy

## Abstract

Journal impact factors were devised to qualify and compare university library holdings but are frequently repurposed for use in ranking applications, research papers, and even individual applicants in mycology and beyond. The widely held assumption that mycological studies published in journals with high impact factors add more to systematic mycology than studies published in journals without high impact factors nevertheless lacks evidential underpinning. The present study uses the species hypothesis system of the UNITE database for molecular identification of fungi and other eukaryotes to trace the publication history and impact factor of sequences uncovering new fungal species hypotheses. The data show that journal impact factors are poor predictors of discovery potential in systematic mycology. There is no clear relationship between journal impact factor and the discovery of new species hypotheses for the years 2000–2021. On the contrary, we found journals with low, and even no, impact factor to account for substantial parts of the species hypothesis landscape, often discovering new fungal taxa that are only later picked up by journals with high impact factors. Funding agencies and hiring committees that insist on upholding journal impact factors as a central funding and recruitment criterion in systematic mycology should consider using indicators such as research quality, productivity, outreach activities, review services for scientific journals, and teaching ability directly rather than using publication in high impact factor journals as a proxy for these indicators.

## ﻿Introduction

The concept of journal impact factors (IFs) was introduced in the 1970s as a means to qualify and compare university library holdings (Garfield 2006; Casadevall and Fang 2014). It was argued that since a journal’s IF is computed as the average number of recent citations to papers recently published in that journal, the IF indicates the relative importance of that journal. IFs could thus be used as guidance for cost-efficient trimming of library holdings – and, by computing average IFs for entire libraries, for ranking libraries according to the perceived importance of their holdings. By and large, the IF concept was well received by the librarian community by virtue of offering some degree of objectivity in what up to that point had been a signally subjective enterprise (Archambault and Larivière 2009). However, it did not take long before the IF concept was invested with meaning and significance far beyond its intended jurisdiction and saw use to rank individual research papers and even researchers according to perceived importance (Simons 2008). A researcher’s average or cumulative IF could be used, many hiring committees and research funding agencies started to argue, to rank candidates or applicants in a reasonably objective, and above all time- and cost-efficient, manner. Heavily laden with mystical import, the IF concept soon took a firm grip on the research landscape (McKiernan et al. 2019).

Over time, parts of the scientific community warmed to the idea that IFs may be an unwarrantably simplistic predictor of past and future research performance (Dong et al. 2005; Krell 2000; Lăzăroiu 2013). Indeed, there are countless examples of pivotal research papers that were published in journals with no, or low, or moderate impact factors – and substandard, faulty, or downright fraudulent papers published in high-IF journals (Trikalinos et al. 2008; Brembs 2018). But still, in the year 2024, the use of IFs as indicators of scientific performance and potential holds significant traction in the scientific community. Many of the present authors regularly serve in various evaluation committees for promotions, academic positions, and research grants in systematic mycology and beyond. In this capacity we are typically asked to rank applicants or applications in order of relevance to the matter at hand. We’re instructed – and happy – to consider a multitude of parameters, including research performance, previous grants, stays abroad, time spent on parental leave, teaching, supervision, review services, and outreach. But often enough, when the committee finally convenes to negotiate a joint position, IFs tend to surface as the most decisive – and sometimes the only – parameter of relevance. The oracular emphasis laid on IFs at the expense of all other parameters is a perennial source of amazement to us. It effectively punishes researchers and research groups who ever spent any time doing anything other than maximizing IFs (Rushforth and de Rijcke 2015; Johann et al. 2024). But is it really the case that mycological discovery scales in a linear way with impact factor so that by choosing the application/applicant with the highest impact factor statistics, we get the most mycology for the money? Several of us have struggled to look ourselves in the mirror following committee meetings of this kind.

In the context of systematic mycology, down-prioritizing researchers and research groups without a strong track record of high-IF publications would make sense if, indeed, low-IF publications and no-IF publications do not contribute much, or anything, to systematic mycology. Conversely, if it is the case that also (or even primarily) low- and no-IF publications make substantial contributions to this field, then the usefulness of IFs as a decisive indicator in systematic mycology would be illusory and, in fact, directly counterproductive. Is there data to form some sort of evidential underpinning for the contribution of IFs to systematic mycology? We argue that there is. The UNITE database for molecular identification of eukaryotes clusters all public, full-length fungal barcode (nuclear ribosomal internal transcribed spacer, or ITS) sequences in the International Nucleotide Sequence Database Collaboration (Arita et al. 2021) into roughly species-level entities referred to as species hypotheses (SHs; Abarenkov et al. 2024). These SHs can be thought of as digital twins of the underlying species, and meticulous record and rich metadata are kept for all sequences in each SH, including the publication history of each sequence. Theory can thus be pitted against experiment by querying the IF of the constituent sequences of SHs. Is it really the case that new species of fungi are primarily found in high-IF publications, for instance? And is the trend of IFs versus mycosystematical discovery linear, just like parts of the mycological community seem so happy to assume? Do scientific outlets without formal IFs really contribute nothing to mycology? The present study sets out to assess whether the reliance put on IFs in systematic mycology holds up to empirical scrutiny. Our results suggest that the use of IFs as arbiters of scientific quality and discovery potential in systematic mycology is not consistent with the image of rationality that we feel systematic mycology should seek to project.

## ﻿Materials and methods

The full flow of operation behind the UNITE database is described elsewhere (Kõljalg et al. 2013, 2020; Abarenkov et al. 2024). In brief, UNITE clusters the ITS sequences of the International Nucleotide Sequence Database Collaboration (INSDC) jointly with UNITE-contributed environmental DNA (eDNA, DNA obtained from mixed/bulk samples) ITS sequences into species hypotheses at distance thresholds 0.5% through to 3.0% in steps of 0.5%. These operational taxonomic units can be thought of as entities roughly at the species level. The sequences and the SHs are available for web-based interaction as well as for download in various formats (https://unite.ut.ee/repository.php).

The 1,258,182 Sanger sequencing-derived sequences of UNITE eukaryotic release 10 were found to be distributed across 182,847 SHs at the default 1.5% sequence dissimilarity level. We targeted sequences submitted to the INSDC in the interval 2000–2021. All 43,057 non-singleton SHs whose first (earliest date of INSDC deposition) sequence was annotated (by default or by subsequent third-party sequence annotation) as fungal were targeted. These SHs comprised a total of 506,103 sequences. All other SHs were considered to represent non-fungal eukaryotes and are not treated any further in this study. We examined all sequences computationally for information on publication of origin. A total of 23,710 (55.1%) fungal SHs were first discovered through a sequence for which a published study of origin was specified in INSDC, leaving 19,347 (44.9%) of the initial-SH-discovery sequences with a publication status of the “Unpublished” or “Direct submission” kind. Some proportion of these seemingly unpublished sequences can be expected to be published but not updated with publication information in INSDC (Durkin et al. 2020). We thus subjected all 19,347 such seemingly unpublished sequences to manual Google and Google Scholar searches to see if they in fact had been published. In these queries, we used the INSDC accession number, the author names, and the title of the study (as available).

Official journal impact factors were compiled from ISI Web of Science for the period 2000–2021 for all journals sporting sequences in all SHs deemed to be fungal. The annual median impact factor for mycology was inferred from the 33 mycological journals in ISI’s journal category “Mycology”. Each sequence was assigned the impact factor of its outlet and the year of publication in the IF window 2000–2021. Two alternative approaches were adopted for sequences published in an outlet without a formal IF for the year of publication. In the “strict median” approach, they were not assigned any IF value and were excluded from estimates of median IFs. In the “relaxed median” approach, they were assigned an IF of 0.0 and were included in estimates of median IFs. IFs were considered down to the three decimal digits supported by ISI Web of Science. As a baseline, we also analyzed all formal fungal species descriptions 2000–2021 for impact factor using GBIF (https://www.gbif.org/) and MycoBank (Robert et al. 2013).

## ﻿Results

We found 43,057 non-singleton UNITE SHs to be fungal. The first (oldest) sequence in each such SH was examined for publication information. More than half (23,710; 55.1%) of these were found to be annotated to publication of origin, leaving 19,347 (44.9%) of the “Unpublished” and “Direct submission” kind. We were able to track down 10,203 (52.7%) of these to a published study of origin, giving us a final dataset of 33,913 published sequences, each representing a first, initial discovery of an SH. These 33,913 sequences were found to come from 6,878 studies. Sequences only released through B.Sc./M.Sc./Ph.D. theses were scored as unpublished. The unpublished sequences are not considered any further in this study. The SH-derived sequences of the study were found to have been published in well over 1,500 different journals and outlets, ranging from top-tier international journals with an IF of over 30 to what seemed to be regional or even local journals without an online presence.

**Figure 1.**
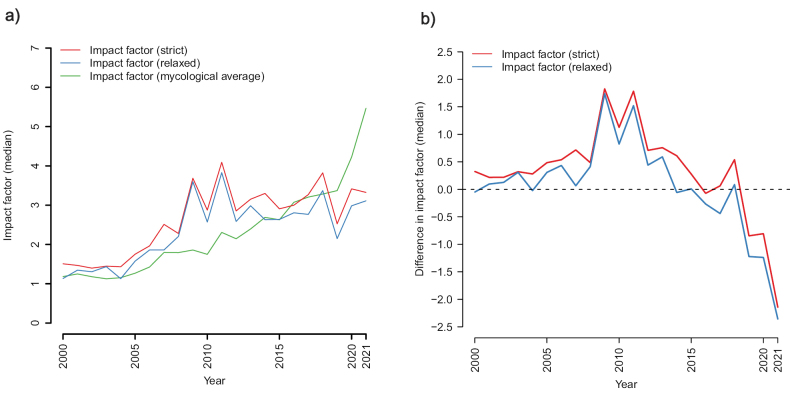
**a** the median impact factor of initial discoveries of UNITE species hypotheses (SHs). For the red curve, only sequences published in a journal with a formal impact factor from the year of publication were included in the calculation. For the blue curve, also sequences published in journals without a formal impact factor from the year of publication were included with their impact factor set to 0.0. The green curve shows the average impact factor of the journals in ISI’s category “Mycology” over time **b** the median impact factor of initial discoveries of SHs visualized as the difference in IF from the median mycological IF (dashed line) over time. The post-2015 drop in relative impact factor is presumably explained by the trend of increasing IFs in ISI’s category “Mycology” over time and mycologists’ apparent struggle to take advantage of this trend when publishing.

**Figure 2.**
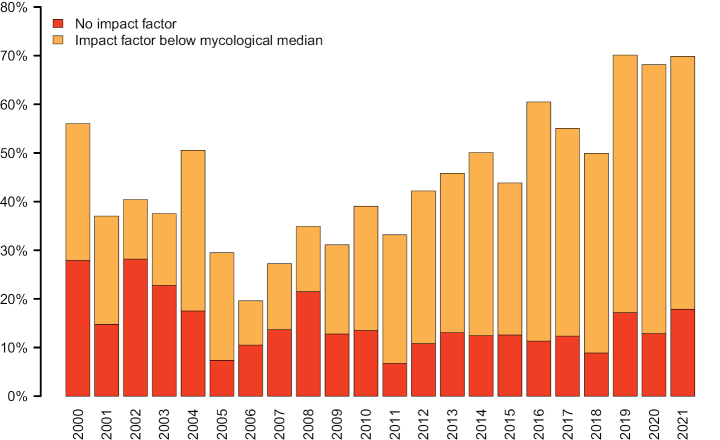
The proportion of SHs whose initial discovery was reported in a journal without a formal IF (red) or with an IF below the mycological median (orange) over time.

**Figure 3.**
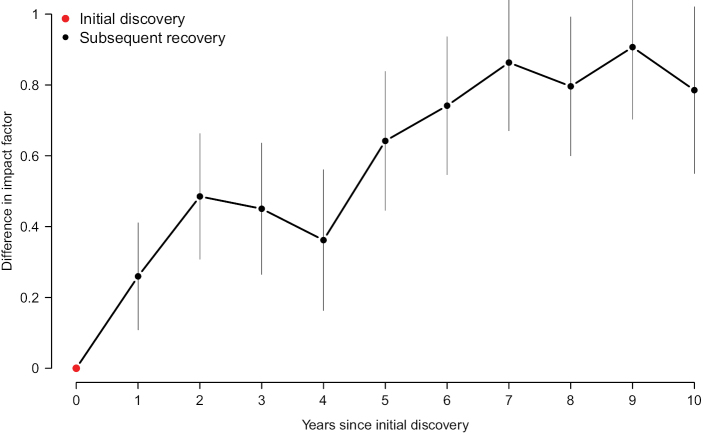
The median IFs of sequences inside SHs over time. SHs were discovered at year 0. The SHs were then inspected for subsequent recoveries in journals with a formal IF, and the value plotted is the median of all such recoveries. To account for the trend of increasing IFs in mycology, the data was normalized by subtracting the median impact factor of the journals in ISI’s category “Mycology”. The unit of the y axis is difference in IF. The error bars show 95% confidence intervals.

In total, 28,662 (84.5%) of the sequences that were the first to evince a new SH discovery were published in a scientific journal with a formal IF that year, leaving 5,251 (15.5%) of the sequences published in an outlet without. Out of those 5,251 sequences, 2,223 (42.3%) were published in a journal that did not have a formal IF at the time of publication, but that eventually obtained one after an average of 4.7 years, leaving 3,028 (57.7%) of the without-IF-sequences published in an outlet that never had a formal IF (2000–2021). The *strict* and *relaxed* median IFs of sequences discovering new SHs over time are displayed in Fig. 1a. Fig. 1b visualizes the difference between the strict/relaxed approaches and the median IF in mycology. Fig. 2 shows the proportion of SHs whose initial discovery was reported in a journal without a formal IF, and with an IF below the mycological median, respectively, over time. Fig. 3 provides a window on the IF trend inside SHs by plotting the IFs of subsequent recoveries of the SH following its initial discovery. To account for the trend of increasing IFs in mycology over time, the data in Fig. 3 was normalized by subtracting the median impact factor of the journals in ISI’s category “Mycology”.

11,386 (33.6%) SHs contained sequences that were released through two or more distinct studies, all of which either lacked a formal IF or had an IF below the median mycological IF the year of publication. Similarly, 2,048 (6.0%) SHs were found to be known from two or more distinct studies, all of which had an IF above the median mycological IF the year of publication. 313 (0.9%) SHs were found to be known from two or more distinct studies, none of which were published in an outlet with a formal IF. In analogy, 12,477 (36.8%) SHs were found to be known from two or more distinct studies, all of which were published in an outlet with a formal IF at the year of publication. 1,260 (3.7%) non-singleton SHs were recovered both from journals with and without formal IFs. The results of the analysis of the impact factors of formal fungal species descriptions 2000–2021 are given in Suppl. material 1.

## ﻿Discussion

The present study examined the relation of journal IFs to discovery potential in systematic mycology. Our results are largely dispiriting – there seems to be no meaningful correlation between IFs and mycosystematical discovery potential as measured as the discovery of new SHs in UNITE. On the contrary, at least in systematic mycology, journal IFs come across as a concept divested of meaning, or at least the meaning ascribed to it in the committee meetings that many of the present authors regularly attend. For instance, for the last 10 years, the majority of new SHs were first reported from journals with an IF below the median mycological IF in a trend that is accentuated over time (Fig. 2). Similarly, formal description of species seems to be a perpetually below-median exercise (Suppl. material 1). In some sense this indicates that journals with an IF above the mycological median do not play an important role in systematic mycology, yet these high-IF journals are what we tend to put a premium on in mycosystematical committees, at least in our experience. The non-trivial proportion of SHs recovered only from no-IF and below-median-IF studies suggests that mycological research traditions and choices of taxonomic target groups differ widely – in fact, disparately – across and among those who study fungi in one capacity or another. Indeed, to some extent, different groups of fungi are studied in no/below-median-IF journals compared to above-median-IF journals. Suppressing mycological research published in no/low-IF outlets is thus tantamount to advocating a paraphyletic view of the fungal kingdom. Such a stance does not blend well with contemporary phylogenetic thinking, where wide and representative taxon sampling is identified as a non-negotiable cornerstone (e.g., Heath et al. 2008).

In an IF-centred world, important mycological findings would be announced in high-IF journals, and those results would only later trickle down and be subsumed into studies published in journals of lesser, or no, IFs. Our results take umbrage with such a contention (Fig. 3). Indeed, publications in high-IF journals seem to draw from the results of publications that were not published in high-IF journals in a way not usually considered in the committee meetings we attend. Figs 1–3 and Suppl. material 1 jointly suggest that the majority of non-trivial discoveries in systematic mycology – particularly in recent years – are being presented in journals that can be called “below average” or even “objectionable” in the sense of lacking a formal IF altogether. That makes – it seems to us – the practice of prioritizing “above average” journal publications in committee situations inimical to systematic mycology. Clearly, journal IFs and species discovery make uneasy bedfellows. The discovery and description of new species are essential for laying the groundwork for scientific progress, yet they do not necessarily resonate well with the short timeframe for IFs and the criteria used by high-IF journals for publication.

Our results do not necessarily suggest that the mycological community should prioritize low/no-IF researchers and research teams, but rather that IFs are a superficially deep, but deeply superficial, measure of mycosystematical discovery potential. If it, indeed, is mycosystematical discovery potential that we wish to promote, then time’s provision of further and better particulars seems to call for abandoning oversimplified shortcuts in the assessment of a researcher’s previous production. Maybe, in fact, there are no shortcuts (Bertuzzi and Drubin 2013). Maybe committees really have to go through a few of the applicants’ main papers in detail to assess the quality and the scientific explanatory power of their findings. Maybe the committee really needs to examine and compare the citations to each individual paper to further quantify and qualify the import of those papers on mycology. And maybe the proposed research project will have to be given more than fleeting attention after all. That would clearly be a very time-consuming approach – presumably a horrifying thought to many, the present authors included. This is, nevertheless, what our results seem to suggest.

Ranking candidates based on IFs furthermore perpetuates the ‘Matthew effect’ whereby candidates who happen to publish in high impact journals early in their career accrue more recognition and cumulative advantage relative to other candidates (Petersen et al. 2011; Rushforth and de Rijcke 2015). This is problematic when candidates have early career advantages not directly related to their research potential. Given many institutions’ commitments to Diversity, Equity, and Inclusion (DEI), the elimination of IF-bias in highly specialized fields where this metric has been shown to be a poor indicator of research contributions should be a priority. The self-correcting nature of science reflects openness to revision when new data come to light, something that we feel should apply also to the evaluation of science. Considering our work and others, committees that insist on making decisions that are heavily weighted by IFs should also consider the risk of bias entrenched in current processes (Boutron et al. 2023).

This study makes the simplification to define “systematic mycology” as the field that discovers and describes new species and groups of fungi – which is what the present study quantifies. We are well aware that systematic mycology covers more than just that, and that the discovery of new SHs in UNITE and formal description of species do not do full justice to the discipline. At the same time, it would seem like a stretch to argue that the discovery and formal description of new species and groups of fungi, unlike all other aspects of systematic mycology, scale poorly to IFs. Instead, we hypothesize that our data speak reasonably well for all of systematic mycology in arguing against the use of IFs as a decisive indicator in systematic mycology. Our study made heavy use of the UNITE SH system, which is based on the formal fungal barcode, the nuclear ribosomal ITS region (Schoch et al. 2012). While we agree that the ITS region is by all accounts the best choice of a singular fungal barcode, it is nevertheless a genetic marker that does not reflect species boundaries perfectly throughout the fungal kingdom (Abarenkov et al. 2016). We used the dynamic SH release of UNITE in an attempt at avoiding the use of static similarity thresholds for automated designations of operational taxonomic units, but it is inevitable that some degree of taxonomic artificiality marks our results (Nilsson et al. 2019).

Our approach was to some extent haunted by missing data – at the onset of the project, a full 19,347 (44.9%) of the sequences representing initial discoveries of species hypotheses were not annotated with a study of origin. We spent more than three months trying to restore this information, but we often found ourselves struggling with journals without a digital presence, journals in other languages than the present set of co-authors had access to, special characters, conflicting information, and the sheer magnitude of the task at hand. In the end, we were able to restore the publication information for 10,203 sequences, reducing the share of “unpublished” sequences from 49.7% to 21.2%. We find it remarkable that upwards of half of the public fungal barcode sequences older than two years were un-annotated to begin with in this regard. Durkin et al. (2020) provided data to suggest that systematic mycology does not maximize its scientific and outreach potential, and the present study lends further weight to those claims. Another source of bias in our results comes from our decision to target only the INSDC and Sanger-sequencing-derived sequences in UNITE. In addition to these sequences, UNITE also features five very large metabarcoding datasets published in high-profile journals (Nilsson et al. 2023). We felt that if we were to also include metabarcoding studies in our approach, then we would have to include not just five high-profile – but all extant – metabarcoding studies to get an unbiased view. However, there is no centralized resource where all metabarcoding datasets are available in an accessible way, and the prospects for clustering billions or even trillions of sequences into the UNITE SH system would furthermore have been bleak. The present paper thus reflects the Sanger sequencing view of systematic mycology.

## ﻿Conclusion

Mycologists regularly report feeling compelled to publish in high-IF journals by virtue of professionalism. Our data suggest that if we by professionalism mean keeping the best interest of systematic mycology in mind, then journal IFs are at a particular risk of misinterpretation – and are regularly ascribed a weight that endangers progress in the field. We eagerly anticipate a future where applications and candidates are assessed in a more integrative way than simple summary metrics obtained from journal IFs, and where mycological contributions are quantified in a way agnostic of the very journal in which they happened to be published. Our non-trivial experience of serving in various evaluation committees is dispiriting in this regard, painting a bleak picture for the future of systematic mycology in a time when the understanding of fungal diversity is more important than ever.

## ﻿Additional information

### Author ORCIDs

R. Henrik Nilsson https://orcid.org/0000-0002-8052-0107

Arnold Tobias Jansson https://orcid.org/0000-0002-9100-9814

Christian Wurzbacher https://orcid.org/0000-0001-7418-0831

Sten Anslan https://orcid.org/0000-0002-2299-454X

Pauline Belford https://orcid.org/0009-0005-5297-483X

Natàlia Corcoll https://orcid.org/0000-0002-1660-9422

Masoomeh Ghobad-Nejhad https://orcid.org/0000-0002-7807-4187

Mikael Gustavsson https://orcid.org/0000-0003-0635-8321

Daniela Gómez-Martínez https://orcid.org/0000-0001-8834-8930

Faheema Kalsoom Khan https://orcid.org/0000-0002-4891-953X

Maryia Khomich https://orcid.org/0000-0002-6840-5739

Charlotte Lennartsdotter https://orcid.org/0009-0008-2780-4881

David Lund https://orcid.org/0000-0003-4075-7529

Breyten Van Der Merwe https://orcid.org/0000-0003-0546-5619

Vladimir Mikryukov https://orcid.org/0000-0003-2786-2690

Teresita M. Porter https://orcid.org/0000-0002-0227-6874

Sergei Põlme https://orcid.org/0000-0002-9658-1166

Alice Retter https://orcid.org/0009-0004-3758-4123

Marisol Sanchez-Garcia https://orcid.org/0000-0002-0635-6281

Sten Svantesson https://orcid.org/0000-0003-3435-3659

Patrik Svedberg https://orcid.org/0000-0001-6622-5366

Duong Vu https://orcid.org/0000-0001-7960-2765

Martin Ryberg https://orcid.org/0000-0002-6795-4349

Kessy Abarenkov https://orcid.org/0000-0001-5526-4845

Erik Kristiansson https://orcid.org/0000-0002-8609-2414

### Data availability

All of the data that support the findings of this study are available in the main text or Supplementary Information.

## Supplementary materials

Supplementary material 1 Supplementary item Data type pdf Explanation note **a** the median impact factor of formal fungal species descriptions 2000-2021. For the red curve, only descriptions published in a journal with a formal impact factor from the year of publication were included in the calculation. For the blue curve, also descriptions published in journals without a formal impact factor from the year of publication were included with their impact factor set to 0.0. The green curve shows the average impact factor of the journals in ISI’s category “Mycology” over time **b** the median impact factor of formal species descriptions visualized as the difference in IF from the median mycological IF (dashed line) over time **c** the proportion of formal description of species in journals without a formal IF (red) or with an IF below the mycological median (orange) over time. https://binary.pensoft.net/file/1178929This dataset is made available under the Open Database License (http://opendatacommons.org/licenses/odbl/1.0/). The Open Database License (ODbL) is a license agreement intended to allow users to freely share, modify, and use this Dataset while maintaining this same freedom for others, provided that the original source and author(s) are credited.R. Henrik Nilsson, Arnold Tobias Jansson, Christian Wurzbacher, Sten Anslan, Pauline Belford, Natàlia Corcoll, Alexandra Dombrowski, Masoomeh Ghobad-Nejhad, Mikael Gustavsson, Daniela Gómez-Martínez, Faheema Kalsoom Khan, Maryia Khomich, Charlotte Lennartsdotter, David Lund, Breyten Van Der Merwe, Vladimir Mikryukov, Marko Peterson, Teresita M. Porter, Sergei Põlme, Alice Retter, Marisol Sanchez-Garcia, Sten Svantesson, Patrik Svedberg, Duong Vu, Martin Ryberg, Kessy Abarenkov, Erik Kristiansson
