# Supplementary figures and images for: ﻿20 years of bibliometric data illustrates a lack of concordance between journal impact factor and fungal species discovery in systematic mycology
Source: MycoKeys. 2024 Nov 20;110:273–85. doi: 10.3897/mycokeys.110.136048 (PMC11603103; doi:10.3897/mycokeys.110.136048)

Supplementary figure 1a

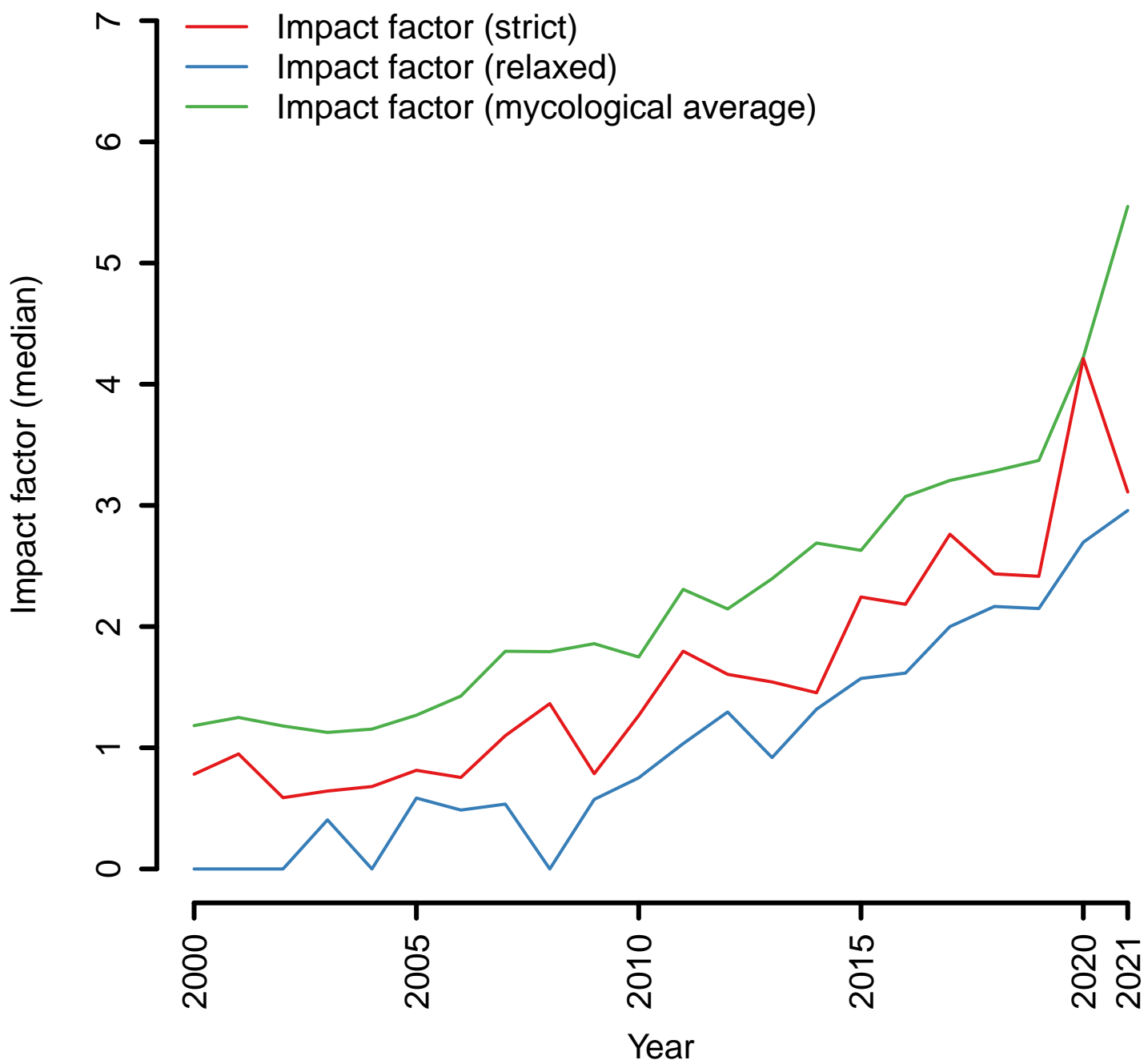

Supplementary figure 1b

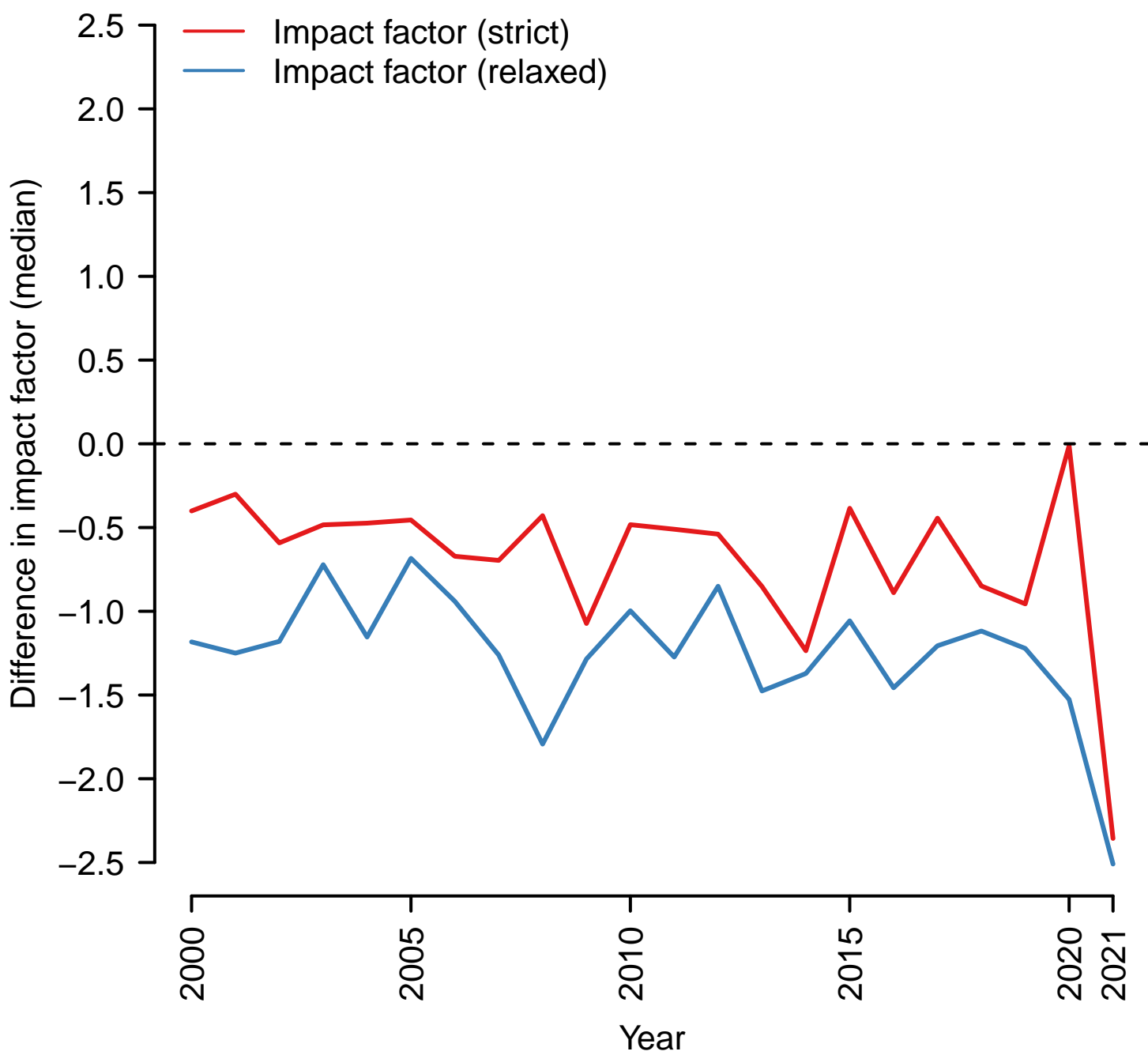

Supplementary figure 1c

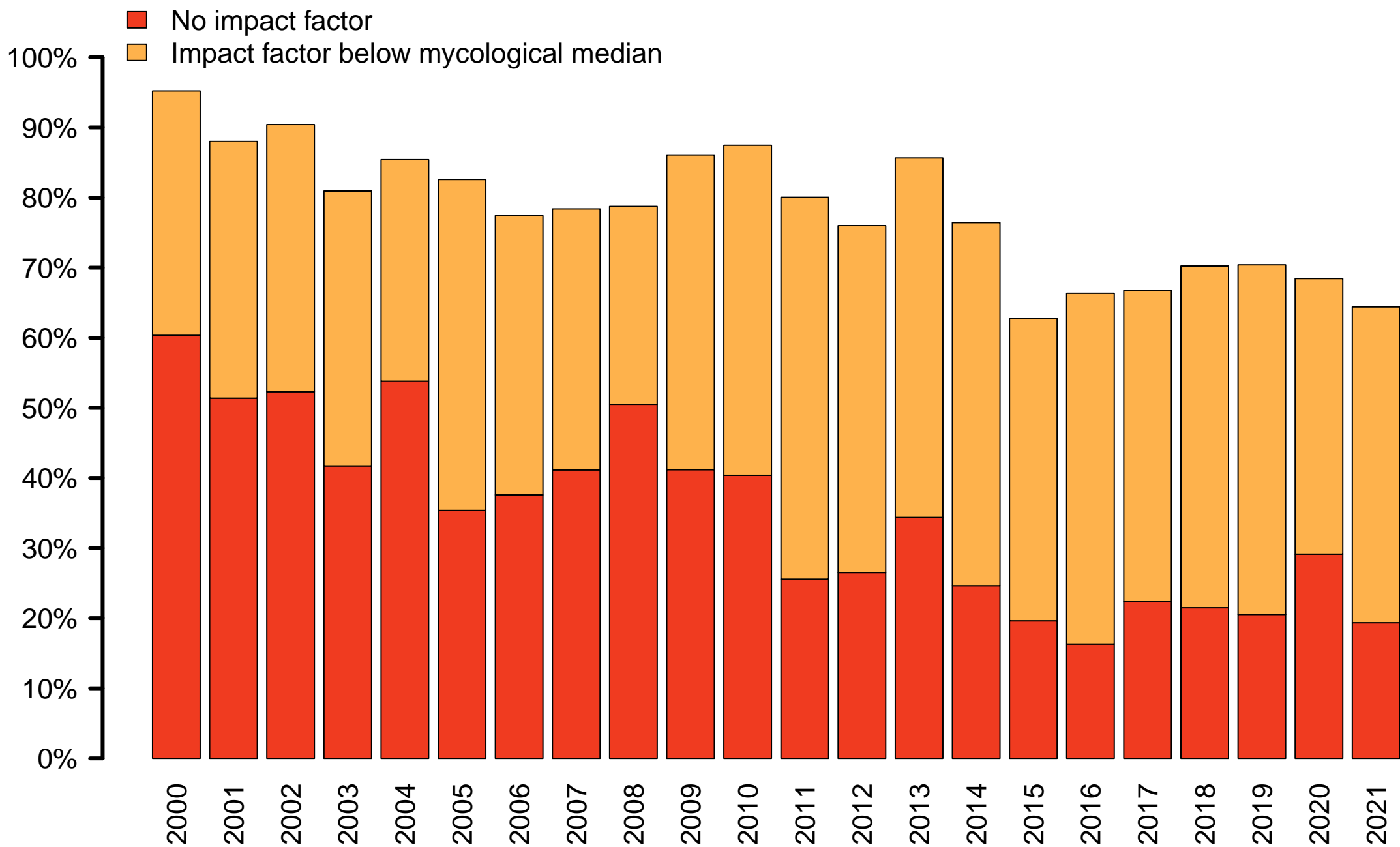

Supplement: Supplementary material 1 — Supplementary item [file mycokeys-110-273-s001.pdf]
